# Microglia and neurons in the hippocampus of migratory sandpipers

**DOI:** 10.1590/1414-431X20155005

**Published:** 2015-11-17

**Authors:** C.G. Diniz, N.G.M. Magalhães, A.A. Sousa, C. Santos, D.G. Diniz, C.M. Lima, M.A. Oliveira, D.C. Paulo, P.D.C. Pereira, D.F. Sherry, C.W. Picanço-Diniz

**Affiliations:** 1 Laboratório de Investigações em Neurodegeneração e Infecção, Hospital Universitário João de Barros Barreto, Instituto de Ciências Biológicas, Universidade Federal do Pará, Belém, PA, Brasil; 2 Laboratório de Biologia Molecular e Ambiental, Instituto Federal de Educação, Ciência e Tecnologia do Pará, Bragança, PA, Brasil; 3 Department of Psychology Advanced Facility for Avian Research, University of Western Ontario, London, Ontario, Canada

**Keywords:** Hippocampus, Neurons, Microglia, Shorebirds, Stereology, Morphometry

## Abstract

The semipalmated sandpiper *Calidris pusilla* and the spotted sandpiper *Actitis macularia* are long- and short-distance migrants, respectively. *C. pusilla* breeds in the sub-arctic and mid-arctic tundra of Canada and Alaska and winters on the north and east coasts of South America. *A. macularia* breeds in a broad distribution across most of North America from the treeline to the southern United States. It winters in the southern United States, and Central and South America. The autumn migration route of *C. pusilla* includes a non-stop flight over the Atlantic Ocean, whereas autumn route of *A. macularia* is largely over land. Because of this difference in their migratory paths and the visuo-spatial recognition tasks involved, we hypothesized that hippocampal volume and neuronal and glial numbers would differ between these two species. *A. macularia* did not differ from *C. pusilla* in the total number of hippocampal neurons, but the species had a larger hippocampal formation and more hippocampal microglia. It remains to be investigated whether these differences indicate interspecies differences or neural specializations associated with different strategies of orientation and navigation.

## Introduction

There is growing evidence that cognitive abilities are influenced by specific ecological conditions to which animals are exposed, and migratory birds are a good example of this (1). Indeed, a significant number of migrants return to the same breeding, wintering, and stopover sites every year (2-5). This observation suggests that migrants have evolved learning and long-term spatial memory abilities that are integrated into a navigational system for repeatedly locating breeding, wintering, and stopover sites (6). The hippocampus is involved in spatial memory in birds and mammals and, hence, may be important in shorebirds for recalling landmarks and migratory routes.

Neuroanatomical differences in the hippocampal formation have been identified when comparing migratory and non-migratory bird species (7). However, investigations focused on the neurobiological basis of hippocampal plasticity in birds have largely been directed at volumetric changes and numerical estimates of hippocampal neurogenesis, with only a few reports dedicated to examining the relationship between glial cells and hippocampal function (8,9). One such study examined variations in glial cells numbers in birds that store and retrieve food and measured the effect of environmental influences on the number of hippocampal glia in *Poecile atricapillus* (8). These authors found that animals living freely under the influence of natural environmental pressures tend to have significantly more glial cells than those living in captivity, suggesting that the environment influences the number of glial cells. In addition, they showed that hippocampal volume increases with the number of glial cells, but not with increasing neurogenesis (8).

The classical functional description of microglial cells is a macrophage or macrophage-related cell. However, accumulating evidence suggests that microglia modulate neurotransmission and synaptic plasticity by secreting several soluble factors or by engaging in synaptic remodeling (10,11). No hippocampal comparisons were made between long-distance migratory birds with distinct migration patterns. The sandpiper *Calidris pusilla* is a long-distance migrant with 6 days of non-stop flights of up to 4000 km between key stopover sites (12,13). *C. pusilla* has a narrow band migration pattern and moderate dispersion on spring and summer sites (14), whereas *Actitis macularia* presents a distinctly different migration pattern on broad fronts with many stopover sites (14,15) and a broad dispersion on spring and summer grounds (14). The migration timing of these species is very similar, although the distance is slightly less for *A. macularia* (14).

Because the autumn migration route of *C. pusilla* includes a non-stop flight over the Atlantic Ocean, and the autumn route of *A. macularia* is largely over land, we predicted that the hippocampus of these sandpipers would be different and that this difference could be related to glial numbers and morphology.

Detailed three-dimensional (3D) morphological studies of microglia in the hippocampal formation of birds are not available, and only a single stereological analysis (glia and neurons) has been published (8). Indeed, previous microglial reconstructions were based on two-dimensional reconstructions and included only a few species: pigeon (16), chicken (17), and quail (18), suggesting a conservative morphological pattern in different bird species.

## Material and Methods

Overwintering sandpipers *A. macularia* and *C. pusilla* were collected in January and February on Canela Island in the tropical coastal zone of northern Brazil (00°47′09.07-S and 46°43′11.29-O). All animals (n=4 per species) were captured under license No. 16086-1 from the Brazilian Institute of Environment (IBAMA), the Brazilian federal institution that regulates the use of wild animals in scientific research. All procedures were carried out under the approval of the Institutional Ethics Committee for Animal Experimentation of the Universidade Federal do Pará in accordance with National Institutes of Health (USA) and Brazilian regulations for scientific procedures on animals. All efforts were made to minimize the number and suffering of animals used.

### Perfusion and histology

After an overdose of a mixture of 10 mg/kg xylazine and 100 mg/kg ketamine, all animals were perfused through the heart with saline, followed by aldehyde fixatives (4% paraformaldehyde, 0.1 M phosphate buffer, pH 7.2-7.4). Entire brains were cut into 50-µm-thick coronal sections using a vibratome (Leica, Biosystems, USA) to generate five series of sections. Each section represented a known fixed fraction of the tissue. Therefore, the section sampling fraction (ssf) for stereology was 1/5. All sections were mounted on glass slides coated with an aqueous solution of gelatin (4.5%) and chromium potassium sulfate 4.0%, air-dried at room temperature, dehydrated, and cleared in an alcohol and xylene series.

### Immunohistochemistry

For immunolabeling, free-floating sections were pre-treated with 0.2 M boric acid, pH 9, at 65-70°C for 60 min to improve antigen retrieval, washed in 5% PBS/Triton-X, and incubated in methanol/3% H_2_O_2_. Sections were then immersed for 20 min in 10% normal horse serum and then transferred to the primary antibody (NeuN, MAB377 Chemicon, USA) diluted in phosphate-buffered saline (PBS, 1:1000) and incubated for 3 days at 4°C with gentle and continuous agitation. Washed sections were then incubated overnight in secondary antibody (biotinylated horse anti-mouse, 1:200 in PBS, Vector Laboratories Ltd, USA) followed by immersion in avidin-biotin-peroxidase complex (Vectastain ABC kit; Vector Laboratories ) solution (1:100 in 0.1 M PO_4_ buffer, pH 7.2-7.4), for 60 min as recommended by the suppliers (Vector Laboratories). Sections were washed and reacted to visualize horseradish peroxidase (HRP) enhanced by the glucose-oxidase-DAB-nickel method. We evaluated the specificity of immunohistochemical patterns by omitting the primary antibody (19), which revealed no unspecific labeling. After immunolabeling, all sections were counterstained by cresyl violet.

An alternate series of sections was immunolabeled with a polyclonal antibody specific for ionized calcium-binding adapter molecule 1 to detect microglia and/or macrophages (anti-Iba1, #019-19741; Wako Pure Chemical Industries Ltd., Japan). For immunolabeling, free-floating sections were pre-treated with 0.2 M boric acid, pH 9, at 65-70°C for 60 min to improve antigen retrieval, washed in 5% PBS, immersed for 20 min in 10% casein (Vector Laboratories), and then incubated with anti-Iba1 (2 µg/mL in PBS) diluted in 0.1 M PBS, pH 7.2-7.4, for 3 days at 4°C with gentle and continuous agitation. Washed sections were then incubated overnight with secondary antibody (biotinylated goat anti-rabbit, 1:250 in PBS, Vector Laboratories). Endogenous peroxidases were inactivated by immersing the sections in 3% H_2_O_2_/PBS, then washed in PBS, and transferred to a solution of avidin-biotin-peroxidase complex (Vectastain ABC kit; Vector Laboratories) solution for 1 h. The sections were washed again before incubation in 0.1 M acetate buffer, pH 6.0, for 3 min, and developed in a solution of 0.6 mg/mL diaminobenzidine, 2.5 mg/mL ammonium nickel chloride, and 0.1 mg/mL glucose oxidase. After immunolabeling, all sections were counterstained by cresyl violet.

We defined the sandpiper hippocampal formation as comprising the hippocampus proper and the parahippocampal area. For the hippocampus (Hp), the lateral and ventral limits were defined by the lateral ventricle, the dorsal and caudal limits corresponded to the cerebral surface, the medial limit was defined by the interhemispheric fissure, and the inferior limit was defined by a marked change in cell density in the dorsal-most hippocampal “V” region near the septal area. The parahippocampal area was located dorsal and lateral to the hippocampus, as defined medially by the paraventricular sulcus (20).

### Hippocampal and telencephalon volumes

To measure hippocampal and telencephalon volumes, and the ratio between them, we followed the total telencephalon method as previously described (21). To do so, we used an optical fractionator (StereoInvestigator, MBF Bioscience, USA), a standard stereological method that estimates volumes based on the Cavallieri method (22). The values for statistical analyses were extracted from the Neu-N and IBA-1 series. The telencephalon (telencephalon+hippocampus) was measured beginning from the first tissue section of the telencephalon through the last section of the telencephalon, as previously described (23).

### Microglial morphometry

For each *C. pusilla* and *A. macularia* specimen, 36 microglial cells were digitally reconstructed in three dimensions from hippocampal sections. We used a Nikon Eclipse 80i microscope (Japan), equipped with a motorized stage (MAC6000, Ludl Electronic Products, USA). Images were acquired under oil immersion with a high-resolution using a 100× oil immersion plan fluoride objective (Nikon, NA 1.3, DF=0.19 µm), and a computer running the Neurolucida software (MBF Bioscience Inc.). Only cells with branches that were unequivocally complete were included for 3D analysis (cells were discarded when branches appeared artificially cut or not fully immunolabeled). Terminal branches were typically thin. Microglial cells were selected from both dorsal and ventral hippocampal sections. Although many morphological features were analyzed, we describe here only those for which we found significant differences. Twelve microglial parameters (4 related to the soma and 8 to the microglial branches) were estimated and compared: 1) branch length (µm); 2) surface area (µm^2^); 3) branch volume (µm^3^); 4) segments/mm; 5) tortuosity; 6) fractal dimension (k-dim); 7) base diameter of the primary branch (µm); 8) total number of segments; 9) soma area (µm^2^); 10) soma perimeter; 11) ferret maximum diameter (maximum diameter possible of a shape); and 12) ferret minimum (minimum diameter possible of a shape). All measurements were made with Neurolucida and extracted with the Neuroexplorer software (MBF Bioscience Inc.).

### Microglia and neuronal numbers

After neuronal or microglial selective immunolabeling, we estimated the numbers of NeuN and IBA-1 immunolabeled cells in both *C. pusilla* and *A. macularia*. We used the optical fractionator to determine cell numbers. The optical fractionator is unaffected by histological changes, shrinkage, or damage-induced expansion of tissue (24). Each hippocampal contour from one hemisphere was digitized directly from each section using a 4.0× objective on a Nikon Eclipse 80i microscope equipped with a motorized stage (MAC6000, Ludl Electronic Products). High-power images were acquired under oil immersion, with a high-resolution, 100×, oil immersion plan fluoride objective (Nikon, NA 1.3, DF=0.19 µm), and a computer running the Stereo Investigator software (MBF Bioscience Inc.), which was used to store and analyze the x, y, and z coordinates of the digitized points. We began by screening the complete section from one hemisphere to delineate the hippocampal region on the computer screen. The borders of the hippocampus were defined according to changes identified in the staining pattern of each marker. To unambiguously detect and quantify the objects of interest in the dissector probe, the low-power objective was replaced by a high-resolution, 100×, oil immersion plan fluoride objective (Nikon, NA 1.3, DF=0.19 µm). For each quantification site, the section thickness was carefully assessed using the high-power objective and fine focus of the microscope to define the immediate defocus above (top of section) and below (bottom of section). Because both the thickness and neuronal distribution in the sections were uneven, we estimated the total number of neurons based on the number-weighted section thickness. This number shows the estimated population count determined by the selected series of optical fractionator runs; the section thickness value was used for the number of weighted section thickness (MBF Bioscience). All sampled neurons or microglia that came into focus inside the counting frame were quantified and added to the total marker sample, provided that the cell bodies were entirely within the counting frame or intersected the acceptance line without touching the rejection line. The optical fractionator method determines the number of cells by multiplying the number of objects identified inside each counting box by the values of three ratios: a) the ratio between the number of sections sampled and the total number of sections (section sampling fraction, ssf); b) the ratio of the counting box and the area of the grid (area sampling fraction, asf); and c) the ratio between the height of the counting frame and the section thickness after histological procedures (thickness sampling fraction, tsf). The counting boxes (50×50 µm for Neu-N and 100×100 for IBA-1) were randomly and systematically placed within a grid (350×350 µm for Neu-N and 400×400 µm for IBA-1). The experimental parameters and average counting results for quantified neuronal or microglial markers in each region of interest of one hemisphere are shown in Supplementary Tables S1-S4. These grid sizes were adopted to achieve an acceptable coefficient of error (CE). The calculation of the CE for the total neuronal count in each bird used in the present study adopted the one-stage systematic sampling procedure (Schaeffer CE). The level of acceptable error in the stereological estimations was defined by the ratio between the intrinsic error introduced by the methodology and the variation coefficient. The CE expresses the accuracy of the cell number estimates, and a CE between 0.03 and 0.07 was deemed appropriate for the present study, because variance introduced by the estimation procedure contributes little to the observed group variance (25). The variance introduced by methodological procedures was in most cases less than 50% of the observed group variance giving a ratio CE2/CV2<0.5 (26). There was one exception to this rule, where CE^2^/CV^2^ was 0.09 (Supplementary Table S2) in neuronal counts of *A. macularia,* even though the average of the CE estimates was only 4% and CV=0.13. In this case, a negative coefficient of biological variation (-90.6%) was detected, indicating that Schaeffer’s coefficient of error (CE) was smaller than the coefficient of variation (CV) and that the rule CE^2^/CV^2^<0.05 was neither meaningful nor practical to follow (26).

### Photomicrography

For photomicrographs, we used a digital camera (Microfire, Optronics, USA) coupled to a Nikon Eclipse 80i microscope. Digital photomicrographs were processed using Adobe Photoshop software; scaling and adjustment of brightness and contrast levels were applied to the entire image. To illustrate the average number of microglia from each species, we selected a 3D reconstruction of microglia with morphometric values closest to the mean number of corresponding features of each species.

## Results

**Figure 1:**
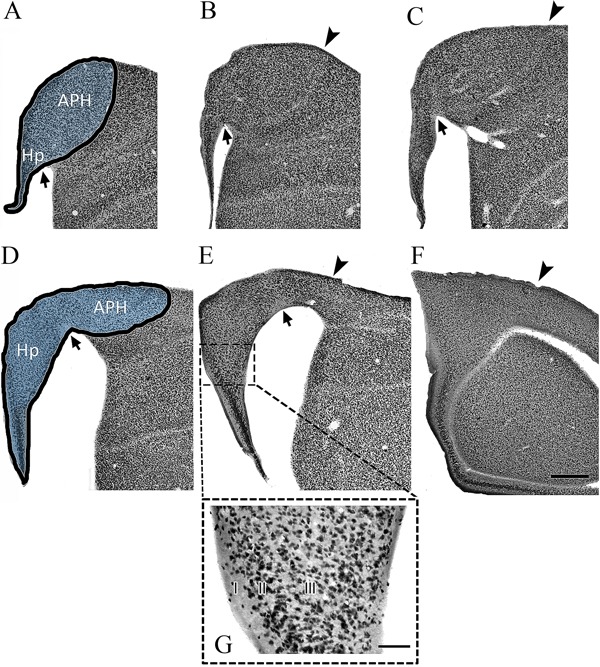
Hipocampal neurons of *Calidris pusilla*. Coronal series of NeuN-immunolabeled sections of the *C. pusilla*hippocampal formation. The left to right sequence is from the frontal to the occipital pole of the hippocampal formation. In the first sections of the top and bottom rows, the dark line defines the area of interest. The arrowheads indicate limits of the area of interest. The arrows indicate the paraventricular sulcus. APH: parahippocampal area; Hp: hippocampus. Scale bar: 500 µm. Photomicrography with 10× magnification of the ventral region of the dorsomedial hippocampus of *C. pusilla*shows three distinct layers: layer I contains a few scattered neurons; layer II is formed by two or three rows of densely packed neuronal cell bodies; layer III contains a less compact more scattered arrangement of neurons. Scale bar: 100 µm.

**Figure 2:**
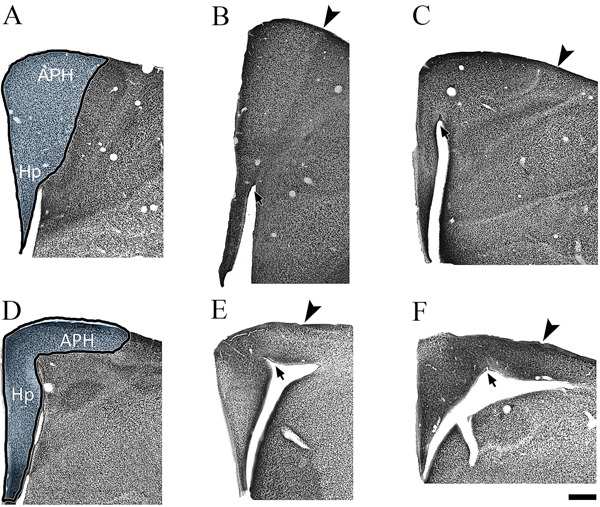
Hippocampal neurons of *Actitis macularia*. Coronal series of NeuN-immunolabeled sections of the *A. macularia*hippocampal formation. The left to right sequence is from the frontal to the occipital pole of the hippocampal formation. In the first sections of the top and bottom rows, the dark line defines the area of interest. The arrowheads indicate limits of the area of interest. The arrows indicate the paraventricular sulcus. APH: parahippocampal area; Hp: hippocampus. Scale bar: 500 µm.

Figures 1 and Figure 2 illustrate the hippocampal formation in *C. pusilla* and *A. macularia* from a series of coronal sections immunolabeled for NeuN. The hippocampal formation in both species comprises two distinct regions: a V-shaped medial region corresponding to the hippocampus proper and the less, well defined, parahippocampal area, located dorsal and laterally to the lateral ventricle. The lateral and medial boundaries of the hippocampal formation are readily identified in the low-power images in Figures 1 and Figures 2.

In *C. pusilla* and *A. macularia,* as expected, the hippocampal area conforms to the general rule for birds (20), that is, wider in the dorsal region at the junction with parahippocampal area and narrow in the ventral portion, near the septum. The parahippocampal area in *C. pusilla* and *A. macularia* is the larger component of the hippocampal formation through most of the rostro-caudal axis. The paraventricular sulcus, indicated by arrows in Figures 1 and 2, separates the hippocampus proper from the parahippocampal area (20). In sections where the paraventricular sulcus is not apparent, the boundary between hippocampal area and parahippocampal area is less clear. In rostral sections, the architectonic boundary between the parahippocampal area and hyperpallium accessorium is also less clear.

The region of the dorsomedial hippocampus of *C. pusilla* and *A. macularia* shows three distinct layers: layer I contains a few scattered neurons; layer II is formed by two or three rows of densely packed neuronal cell bodies; layer III contains a less compact and more scattered arrangement of neurons (See inset in Figure 1).

After selective neuronal immunolabeling, we estimated the numbers of NeuN-immunolabeled cells and detailed results are reported in Supplementary Tables S1 and S2. Stereological parameters, unilateral individual cell numbers, and mean (±SD) numbers of neurons for *C. pusilla* are reported in Supplementary Tables S1 and S3, (n=4) and for *A. macularia* in Supplementary Tables S2 and S4 (n=4).

**Figure 3:**
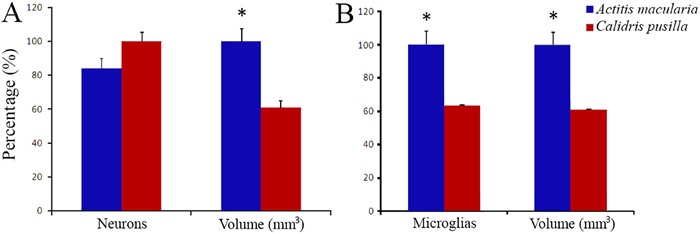
Neurons and microglia in the sandpiper hippocampus. *A*, Number of neurons and volume of the hippocampal formation reported as percentage values in *Actitis macularia* and *Calidris pusilla* (the highest value in each paired data set for the two species was assigned a value of 100%). For absolute numbers, see tables in Supplementary Material. Data are reported as means ± SE and asterisks indicate a statistically significant difference using Student’s *t*-test with P<0.05. *B*, Number of microglia and volume of the hippocampal formation in *A. macularia* and *C. pusilla*. Data are reported as means ± SE and asterisks indicate a statistically significant difference using Student’s *t*-test with P<0.05.

The mean number of neurons in the hippocampal formation was not significantly different between the species (*C. pusilla*: 909,540±138,470 *vs A. macularia*: 764,767±104,962; two-tailed *t*-test for independent samples, P=0.14). Although the volume of the telencephalon (palium+hippocampus) of *A. macularia*(81.7±15.27 mm^3^) was not significantly different from *C. pusilla* (74.2±15.43 mm^3^; two-tailed *t*-test P=0.52), the average hippocampal volume of *A. macularia* (6.11±1.29 mm^3^) was greater than that of *C. pusilla* (3.71±0.74; two-tailed *t*-test, P=0.02). Consequently, the ratio between the volumes of the telencephalon and hippocampus were remarkably different in these species (average ratio in *C. pusilla*: 20.02±2.26 *vs A. macularia*: 13.63±2.46, two-tailed *t*-test, P=0.009). See Figure 3A and Supplementary Tables S1 and S2 for details.

The numbers of microglia of the hippocampus of *C. pusilla* and *A. macularia* are shown in Figure 3B and Supplementary Tables S3 and S4. Unlike neurons, the mean number of microglia in the hippocampus was significantly greater in *A. macularia* (84,112±13,634) than in *C. pusilla*(53,263±12,389; two-tailed *t*-test, P=0.016). The microglial numbers were 37% greater in *A. macularia* than in *C. pusilla* and subsequently a large difference in neuron/microglia ratio (*C. pusilla*: 17.75±3.90 *vs A. macularia*: 9.15±0.83, two-tailed *t*-test, P=0.039). The difference in the telencephalon/hippocampus volume ratio corresponded with the difference in the microglial number ratio, which demonstrated that on average the *C. pusilla* hippocampus is 20 times smaller than the telencephalon, while in *A. macularia* this ratio is only 13.6.

### Microglial morphology

**Figure 4:**
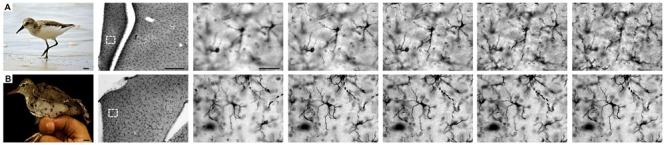
Microscopic appearance of sandpiper microglia. Photomicrographic series at different planes of focus to show microglial immunolabeling in selected sections from the hippocampal formation of *Calidris pusilla* and *Actitis macularia*. The dotted square region at low magnification shows the relative position of microglia illustrated at higher magnification. Scale bars *A*=6 mm; *B*=8 mm; low power=250 µm, high power=25 µm.

Figure 4 shows a series of photomicrographs taken from different focus planes of Iba1-immunolabeled sections to illustrate hippocampal microglia morphology in shorebirds (*C. pusilla* and *A. macularia*, rows A, B).

**Figure 5:**
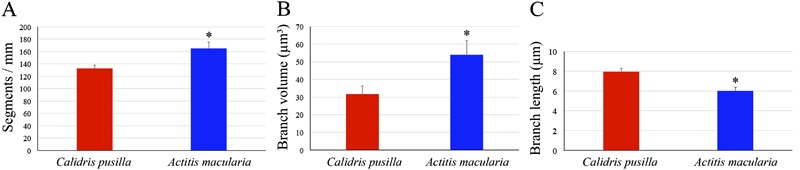
Measurements of sandpiper hippocampus microglial processes. Data are reported as means±SE for 3 distinct microglial morphological measurements, which showed statistically significant differences between shorebirds *Actitis macularia* and *Calidris pusilla* (two-tailed *t*-tests, *P<0.05). Note that, on average, microglial branches from *C. pusilla* have longer and thinner processes and less dense ramifications than those from *A. macularia.*

Figure 5A-C illustrates significant differences in the morphological features of microglial processes in each species. A total of 288 cells, 144 from each species, 36 cells from each individual, were reconstructed and the mean values of each variable are represented in the graphics. On average, microglial branches from *C. pusilla* showed longer and thinner processes and less dense ramifications than those from *A. macularia* (mean branch length±SE: *C. pusilla*: 7.95±0.35 *vs A. macularia*: 6.02±0.35 µm, t=3.95, P=0.001; branch volume: 31.67±4.52 *vs* 53.91±8.14 µm^3^, t=-2.39, P=0.032; segments/mm: 132.51±5.82 *vs*165.07±10.34, t=-2.74, P=0.02).

**Figure 6:**
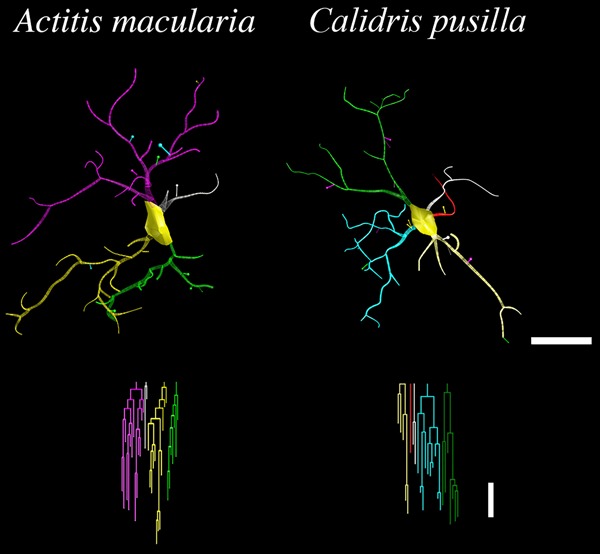
Microglial three-dimensional (3D) reconstructions from sandpiper hippocampus. 3D reconstructions (top) and correspondent dendrograms (bottom) of representative microglia showing differences in morphology between sandpipers. Individual branches are distinctly colored to facilitate examination. The linear dendrograms of microglial arbors show the length of each branch segment displayed to scale; sister branches are horizontally displaced. Branch colors correspond to the 3D reconstructions above. The dendrograms were plotted and analyzed using Neuroexplorer (MBF Bioscience, USA). Dendrograms scale bar=10 µm, 3D reconstructions scale bar=10 µm.

Figure 6 shows 3D reconstructions of microglia from *C. pusilla* and *A. macularia,*with mean branch length, branch volume, and segments/mm closer to mean values of these morphological features illustrated in Figure 5. Note that compared to *A. macularia*, microglial branches from *C. pusilla* had longer, thinner, and less dense ramifications.

## Discussion

By employing selective immunostaining for neurons and microglia, we were able to identify the hippocampal formation boundaries in the sandpipers *C. pusilla* and *A. macularia*, thereby revealing a pattern of organization similar to what was previously described in passerine birds. We found that the number of hippocampal neurons in sandpipers did not significantly differ, whereas the number of microglia in *A. macularia* was 36.7% greater than in *C. pusilla.* The hippocampal volume in *A. macularia* was 39.2% greater than in *C. pusilla* in the absence of any significant difference between these two species in terms of telencephalon size. Microglia processes in *C. pusilla* were longer, thinner, and less numerous than in *A. macularia*.

### Hippocampal formation

**Figure 7:**
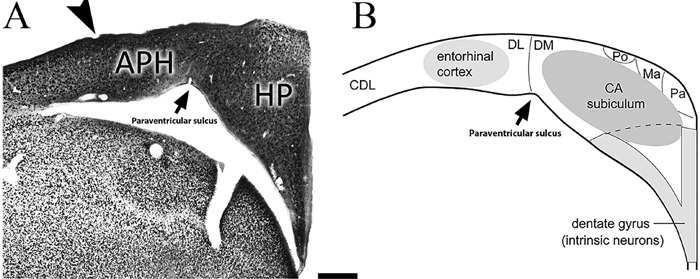
Hypothetical hippocampal homologies of birds and mammals. Left: section of the hippocampal formation of *Actitis macularia* immunolabeled for NeuN. The arrowhead indicates the lateral limit of the parahippocampal area and the arrow indicates the paraventricular sulcus, which is the lateral boundary of the hippocampus. Right: hypothetical homologies between the subregions of the hippocampal formation in mammals and birds. The ventral V-shape on the right (light gray) is comparable to the mammalian dentate gyrus, the dorsomedial area (DM) is comparable to the horn of Ammon (CA), and the subiculum and the dorsolateral (DL) area are homologous to the entorhinal cortex. Other regions include the histologically distinct magnocellular region (Ma), the parvocellular (Pa) and a region poor in cellular elements (Po). APH: parahippocampal area; Hp: hippocampus. Scale: 500 µm. Adapted from Atoji et al. (39).

In the sandpipers we examined, the hippocampal formation of birds (20) shows quite a conserved appearance, retaining features previously proposed to be homologies with the mammalian hippocampal formation (27). Figure 7 compares a coronal section of *A. macularia* with a schematic diagram of a coronal section through the avian hippocampal formation, adopting a previously proposed model (20), to illustrate possible homologies with areas of the mammalian hippocampal formation (27). The hippocampus of *C. pusilla* and *A. macularia* seems to conform to this model. The paraventricular sulcus defines the boundary between the hippocampus and the parahippocampal area, as previously described in other birds (20). The architectonic similarity of *Calidris* and *Actitis* to other birds made it easier to define the limits of the area of interest in our stereological and morphometric assays.

### Sandpiper hippocampal volumes, neurons and microglia

Based on cytoarchitectonics using NeuN immunolabeling, we defined the boundaries of the hippocampal formation and estimated, using selective markers and stereology, the number of neurons and microglia in the hippocampus. *A. macularia*, which migrates overland in a broad front with many stopover sites, had a much larger hippocampus than *C. pusilla,*which makes long-distance flights over the Atlantic Ocean between key stop over sites. The difference in hippocampal size occurred in the absence of any difference between these species in telencephalon size. There was, however, no difference between *A. macularia* and *C. pusilla*in the number of hippocampal neurons. Instead, *A. macularia* had many more hippocampal microglia than *C. pusilla.*

Although *A. macularia* and *C. pusilla* differ in a variety of ways, it may be significant that the hippocampus is larger in the species that probably relies more on visuospatial information for navigation during migration. Navigation during the trans-oceanic flights of *C. pusilla* is less likely to depend on visuospatial information than on geomagnetic compass bearings. The avian hippocampus plays a central role in spatial memory and visuospatial orientation (28-30). Although manipulation of the magnetic field can result in hippocampal activation (31), such manipulations result in activation in many other brain areas of birds, including Cluster N and the brain stem (32-35). Although we cannot exclude the possibility that phylogeny would be sufficient to explain these hippocampi differences, the larger hippocampus of *A. macularia* could also be an adaptation to visuospatial orientation and navigation during migration. The larger hippocampus of *A. macularia* did not contain more neurons than the hippocampus of *C. pusilla,* but instead contained many more glial cells. A previous study showed that food-storing chickadees (*P. atricapillus*) from harsh environments had more hippocampal glial cells than chickadees from milder climates (8). Chickadees from harsh environments also have a larger hippocampus and better spatial memory than chickadees from milder climates (36). These differences in hippocampal size and glial cell number are interpreted as an adaptation to greater reliance on cached food - and a greater reliance on spatial memory to cache and retrieve food - in birds living under harsher conditions. Roth et al. (8) also found that chickadees from two examined populations differed in the number of hippocampal neurons, whereas we found no difference in the number of hippocampal neurons between our sandpipers.

The association between a larger hippocampus and a greater number of hippocampal microglial cells in *A. macularia,* in the absence of any difference in the number of hippocampal neurons, suggests that the relative number of glia alone can influence hippocampal function.

Recent findings revealed that microglia are associated with important physiological functions in learning and memory; they promote learning-related synapse formation through BDNF signaling (10) and after training to learn and remember the spatial location of an object. Results show that microglial-dependent synapse remodeling is evident six hours later in the DG-Mol layer (11). Moreover, we previously demonstrated significant correlations between morphology of dentate gyrus microglia and performances in visuospatial learning and memory task in the monkey brain (37). It has been demonstrated that microglial morphology from phylogenetic distant-related species with lower or higher cognitive performances in hippocampal-dependent tasks are, respectively, less and more ramified (37,38). Taken together, numerical and morphological findings may predict that *A. macularia* will show higher cognitive performances in visuospatial tasks than *C. pusilla*.

Our results suggest that hippocampal microglia may contribute to hippocampus-dependent memory or orientation in some migratory birds. However, *Calidris* and *Actitis* are members of different phylogenetic groups within sandpipers. Therefore, the neuroanatomical differences observed may be due to phylogeny, not adaptation to different migratory strategies. To answer this question comprehensively, future studies will require a much larger-scale comparative analysis of more sandpiper species. Results presented in this paper are the first step in examining species differences among shorebirds.

### Technical limitations

It is difficult to estimate the number of objects in histological sections with stereological methods, because of ambiguities in definition and areas of interest. To reduce the potential sources of error when comparing animal groups, we processed all samples using the same protocols, and all data were collected and analyzed using the same stereological method, software, and hardware. To detect possible variations in the criteria used for identifying objects of interest, we performed checking procedures of the objects of interest by having different investigators count the same regions with the same anti-Iba1 antibody as a microglial marker. As a result, we were able to reduce possible variations associated with non-biological sources to acceptable levels. Additionally, microscopic 3D reconstructions may be affected by mechanical factors associated with vibratome sectioning and the dehydration procedure, which can induce non-uniform shrinkage in the z-axis of the sections. Thus, estimates of modifications in the x/y dimensions during tissue processing cannot be linearly extrapolated to the z dimension. These methodological constraints imposed limitations that must be taken into consideration when interpreting the results of the present study.

## Supplementary Material


