# Supplementary material for: Microglia and neurons in the hippocampus of migratory sandpipers
Source: Braz J Med Biol Res. 2015 Nov 17;49(1):e5005. doi: 10.1590/1414-431X20155005 (PMC4678657; doi:10.1590/1414-431X20155005)
Supplement: Supplementary file 1 [file 1414-431X-bjmbr-1414-431X20155005-supp5005.pdf]

Supplementary Material

Table S1. Stereological results for *Calidris pusilla* NeuN-immunolabeled cells in the left hippocampal formation.

	N	Thickness	CE	Volume (mm^3)
C. pusilla 01	723,297	17.7	0.040	3.869
C. pusilla 02	1,040,384	19.3	0.042	3.794
C. pusilla 03	982,961	18.3	0.037	4.730
C. pusilla 04	891,518	18.1	0.041	2.790
Mean	909,540	18.35	0.040	3.796
SD	138,470	0.681		0.794
CV^2	0.023			
CE^2	0.000075			
CE^2/CV^2	0.0231			
CE^2-CV^2	–99.676			
CVB^2 (%)	–99.985			

$CVB^2 = CV^2 – CE^2$ (CE: Schaeffer coefficient of error; CV: coefficient of variation; CVB: biological variation coefficient). The negative value indicates CE is smaller than CV. The stereological parameters are: A(frame) = 50 × 50 µm, A(x,y step) = 350 × 350 µm; area sampling fraction = 0.02; and section sampling fraction = 0.2.

Table S2. Stereological results for *Actitis macularia* NeuN-immunolabeled cells in the left hippocampal formation.

	N	Thickness	CE	Volume (mm^3)
Am 07 HE	778,022	17,400	0.040	5.294
Am 08 HE	831,024	17,100	0.040	7.582
Am 09 HE	837,535	15,800	0.042	6.752
Am 13 HE	612,484	19,500	0.045	4.953
Mean	764,766	17,450	0.042	6.145
SD	104,962	1,533		1.235
CV^2	0.019			
CE^2	0.002			
CE^2/CV^2	0.094			
CE^2-CV^2	–0.017			
CVB^2 (%)	–90.640			

$CVB^2 = CV^2–CE^2$ (CE: Schaeffer coefficient of error; CV: coefficient of variation; CVB: biological variation coefficient). The negative value indicates CE is smaller than CV. The stereological parameters are: A(frame) = 50×50 µm, A(x,y step) = 350×350 µm; area sampling fraction = 0.02; and section sampling fraction = 0.2.

Table S3. Stereological results for *Calidris pusilla* IBA-1-immunolabeled cells in the left hippocampal formation.

	N	Thickness	CE	Volume (mm^3)
C. pusilla 01	43,937	16	0.045	3.074
C. pusilla 02	41,620	16.7	0.049	3.211
C. pusilla 03	60,749	36.2	0.071	4.687
C. pusilla 04	66,750	27.2	0.041	3.895
Mean	53,263	24.025	0.051	3.717
SD	12,389	9.598		0.740
CV^2	0.054			
CE^2	0.002647			
CE^2/CV^2	0.0489			
CE^2-CV^2	–0.051			
CVB^2 (%)	–95.108			

$CVB^2 = CV^2 – CE^2$ (CE: Schaeffer coefficient of error; CV: coefficient of variation; CVB: biological variation coefficient). The negative value indicates CE is smaller than CV. The stereological parameters are: A(frame) = 100 × 100 μm, A(x,y step) = 400 × 400 μm; area sampling fraction = 0.062; and section sampling fraction = 0.2.

Table S4. Stereological results for *Actitis macularia* IBA-1-immunolabeled cells in the left hippocampal formation.

	N	Thickness	CE	Volume (mm^3)
Am 05	86,327	13.08	0.03	7.16
Am 07	77,772	17.06	0.03	5.74
Am 09	102,051	15.07	0.03	6.53
Am 11	70,299	14.03	0.04	5.31
Mean	84,112	14.81	0.03	6.18
SD	13,634	0.004		0.82
CV^2	0.026			
CE^2	0.0009			
CE^2/CV^2	0.034			
CE^2-CV^2	-0.025			
CVB^2 (%)	-96.574			

$CVB^2 = –CE^2$ (CE: Schaeffer coefficient of error; CV: coefficient of variation; CVB: biological variation coefficient). The negative value indicates CE is smaller than CV. The stereological parameters are: A(frame) = 100 × 100 μm, A(x,y step) = 400 × 400 μm; area sampling fraction = 0.081; and section sampling fraction = 0.2.
